# Duck HMGB2 Mediates Signaling Pathways in the Innate Immunity of Hosts Against Viral Infections

**DOI:** 10.3389/fimmu.2020.572289

**Published:** 2020-10-15

**Authors:** Tingting Zhang, Xinyue Zhang, Zhenhong Sun, Gen Liu, Xiaolan Hou, Liangmeng Wei

**Affiliations:** ^1^Collaborative Innovation Center for the Origin and Control of Emerging Infectious Diseases, College of Basic Medical Sciences, Shandong First Medical University, Tai'an, China; ^2^Shandong Provincial Key Laboratory of Animal Biotechnology and Disease Control and Prevention, Sino-German Cooperative Research Centre for Zoonosis of Animal Origin of Shandong Province, Shandong Agricultural University, Tai'an, China; ^3^Shandong Provincial Engineering Technology Research Center of Animal Disease Control and Prevention, College of Animal Science and Veterinary Medicine, Shandong Agricultural University, Tai'an, China; ^4^Key Laboratory of Precision Oncology of Shandong Higher Education, Institute of Precision Medicine, Jining Medical University, Jining, China

**Keywords:** duck high-mobility group box 2, characterization, subcellular localization, innate immunity, signaling pathway, antiviral activity

## Abstract

High-mobility group box 2 (*HMGB2*) belongs to the HMG-box family that participates in a variety of biologic processes. Recent studies have suggested that HMGB2 plays an important role in the innate immunity of fish. Cherry Valley duck is the main duck bred for meat consumption in China, but there is limited research available on the impact of duck HMGB2 (*duHMGB2*) in antiviral innate immunity. Here, *duHMGB2* genes were first cloned and analyzed from the spleen of Cherry Valley ducks. We show that duHMGB2 is widely distributed in most tissues of healthy ducks, and duHMGB2 was differentially expressed in three organs (the spleen, brain, and lung) of ducks during different viral infections. duHMGB2 is mainly expressed in the nucleus of duck embryo fibroblast (DEF) cells. However, duHMGB2 is released into the cytoplasm after viral infection. DuHMGB2 induced expression of several genes that regulate the immune response. Moreover, duHMGB2 activated and upregulatede transcription factor NF-κB promoter activity. We also used single gene manipulations (knockout or overexpression) to confirm that duHMGB2 can inhibit the replication of duck plague virus, duck Tembusu virus, and the novel duck reovirus in DEF cells. These data show that duHMGB2 can activate the antiviral innate immunity of the host. Thus, *duHMGB2* may be considered an immune adjuvant against infectious diseases in duck.

## Introduction

High-mobility group box 2 *(HMGB2*) belongs to the HMG-box family and its translated protein contains two HMG-box domains and a highly acidic C-terminal tail (1). duHMGB2 is a member of the highly conserved non-histone DNA-binding protein family (2). The HMG-box families are highly conserved and exist widely in most species; *HMGB2* is preferentially expressed in male germ cells. In mammals, the sequence identities of *HMGB1, HMGB2*, and *HMGB3* are more than 80%, but previous experiments have shown that HMGB proteins have independent functions. For example, HMGB1 knockout mice die shortly after birth because of hypoglycemia, but HMGB2 knockout mice are viable, although male mice have reduced fertility (3).

The innate immune system includes a series of cells and related mechanisms that can non-specifically fight foreign infections. Cells of the innate immune system will non-specifically recognize and act on pathogens. Pattern recognition receptors (PRRs) are members of recognition molecules that are mainly located on the surface of innate immune cells and can recognize one or more pathogen-associated molecular patterns (PAMPs) (4). Pattern recognition receptors such as toll-like receptors (TLRs) and retinoic acid-inducible gene I (RIG-I)-like helicases (RLRs) are activated when pathogenic microorganisms invade the organism. Activated receptors can trigger downstream signal transduction pathways to produce type I interferons (IFN-Is) and pro-inflammatory cytokines to induce antiviral responses (5, 6). Previous studies have shown that HMGB proteins are ligands that cause inflammation and can sense nucleic-acid-mediated immune responses. The HMGB proteins function as universal sentinels for nucleic acids and recognize PRRs (discriminative sensing) to activate nucleic-acid-induced innate immune responses (7, 8).

Duck Tembusu virus (DTMUV) is positive-sense single-stranded RNA virus that reduces egg production by 60–80% (9). Duck plague virus (DPV) is an enveloped and double-stranded DNA virus that belongs to the family Herpesviridae. The novel duck reovirus (NDRV) is a non-enveloped and double-stranded RNA virus that belongs to the Reoviridae family. These viruses have contributed to major economic losses in China's duck industry (10, 11). Therefore, an in-depth study of the innate immune mechanism of ducks is particularly important for exploring new models of antiviral infection treatment. We previously showed that duck HMGB1 can activate the host's innate immune response to achieve a broad-spectrum antiviral effect (12). More recent studies have shown that HMGB2 participates in the innate immune response of fish, has immunomodulatory properties, and mediates antiviral innate immune responses (13, 14). Thus, it is meaningful to investigate whether the HMGB2 in ducks also mediates the antiviral innate immune response. Future studies will provide a new potential therapeutic target for duck viral infections.

## Materials and Methods

### Virus Strains and Cells

The DTMUV-FX2010 strain, NDRV, and DPV-GM strains were used in this study as previously described (12). Duck embryo fibroblast (DEF) cells derived from 11-day-old duck embryos were cultured in Dulbecco's modified Eagle's medium (DMEM) (Hyclone, Logan, UT, USA) supplemented with 10% fetal bovine serum (TransGen, Beijing, China). All cells were cultured at 37°C with 5% (v/v) CO_2_.

### Animal Experiments

One-day-old ducks were purchased from a farm near Tai'an, China and bred in the isolator until they were 3 weeks old. Serum samples from 1-day-old ducks were tested by ELISA to verify that all ducks were serologically negative for DTMUV. All ducks were also negative for NDRV and DPV via a quantitative real-time polymerase chain reaction (qRT-PCR). We first randomly divided the ducks into four groups (*n* = 20 for each group). Three of these groups were virus-infected (DTMUV-infected, NDRV-infected, and DPV-infected); and the fourth group was used as a control. In the virus-infected groups, 0.3 mL of virus stock solution (DTMUV, NDRV, and DPV) was intramuscularly injected into each duck. The virus titers of DTMUV, NDRV, and DPV were determined in DEF-infected cells. To facilitate the experimental protocol, the virus titers were diluted to 10^5.3^, 10^4.2^, and 10^6.5^ TCID_50_/mL. The infection doses of DTMUV, NDRV, and DPV were chosen based on previous experimental infection doses (11, 15, 16).

In the control group, each duck was intramuscularly injected with 0.3 mL of physiological saline. Continuous observation was continued for 5 days except for the dead ducks. Five live ducks were euthanized in each group (three virus-infected groups and one control group) on the first, third, and fifth day after inoculation to collect three organs (the spleen, brain, and lung). All tissue samples were stored at −80°C for subsequent extraction of total RNA. The other five ducks from each virus-infected group were used to observe the clinical symptoms and the fatality rate after virus infection. In addition, we selected five 3-week-old healthy ducks to collect lymph, circulation, digestion, respiration, urinary, and central nervous tissues including bursa, spleen, heart, glandular stomach, intestine, trachea, lung, kidney, and brain for total RNA extraction. The expression of duHMGB2 was determined by qRT-PCR.

### Cloning and Analysis of the duHMGB2

Total RNA was extracted from the spleen of healthy ducks using the TRIzol method, and RNA was reverse transcribed into cDNA using the HiScriptRII One Step RT-PCR kit (Vazyme, Nanjing, China). To obtain a full-length coding sequence of duHMGB2, primers (Table 1) were designed according to the predicted sequence (accession number, XM_027456410.1) in Gene Bank. The coding region gene of duHMGB2 was then ligated into pMD19-T vector (Takara, Dalian, China) and sequenced. A phylogenic tree of duHMGB2 was constructed using MEGA 5.1 with the neighbor-joining method.

**Table 1:** Primer sequences used for gene cloning in this study.

**Primer name**	**Primer sequence (5^**′**^-3^**′**^)**	**Purpose**
gHMGB2-F	GCGGAAAACAAGAGGCTCTAA	Gene cloning
gHMGB2-R	AGCCCCTCGGCATACTACTT	
TY-duHMGB2-F	CTTGGTACCGAGCTCGGATCC ATGGGCAAAGGCGACCCAA	Gene cloning
TY-duHMGB2-R	CTCTAGACTCGAGCGGCCGC TCAATGGTGATGGTGATG	

### Plasmid Construction and Cell Transfection

The DNA fragment containing the open reading frame (ORF) of *duHMGB2* was subcloned into pcDNA3.0(+)-Flag to construct the eukaryotic expression plasmid of *duHMGB2*. DEF cells were seeded in 6-well plates, and 2 ug of plasmid was transiently transfected into DEF cells at 80% confluence with Lipofectamine 2000 (Invitrogen, Carlsbad, CA, USA).

### Western Blotting

DEF cells were seeded into 6-well plates, and pcDNA3.0(+)-duHMGB2-Flag and pcDNA3.0(+)-Flag were transfected into DEF cells at 80% confluence. After incubating at 37°C for 24 h, the medium was discarded, washed 3 times with phosphate buffered solution (PBS), and whole cell lysate was prepared using a RIPA lysate containing a protease inhibitor. The sample was diluted with 5 X SDS-PAGE loading buffer and boiled for 10 min. Samples were separated on a 12% SDS-PAGE gel and transferred to a polyvinylidene fluoride (PVDF) membrane (Solarbio, Beijing, China) and blocked with 5% skim milk powder overnight at 4°C. The membrane was incubated with mouse anti-Flag antibody (ProteinTech, Shenzhen, China) for 2 h at 37°C, washed three times with PBST, and incubated with secondary antibody at 37°C for 45 min. After three additional washing steps with PBST, protein bands were visualized with an ECL kit (Bio-Rad, United States).

### Indirect Immunofluorescence Assay

DEF cells were seeded in 24-well culture plates plated with cell climbing slices for the immunofluorescence assay (IFA). The plasmid was transfected into DEF cells and washed twice with PBS after 24 h. DEF cells were fixed with 4% paraformaldehyde for 15 min and permeabilized with 0.1% Triton X-100 for 10 min. The cells were then incubated with mouse anti-Flag antibody (ProteinTech, Shenzhen, China) for 1 h at 37°C and then incubated with fluorescein isothiocyanate (FITC)-anti-mouse IgG (Transgen) for 45 min at 37°C. Finally, the cell climbing slices were removed. After sealing with mounting medium, the cells were observed under a laser scanning confocal microscope.

### Quantitative Real-Time PCR

The treated cell samples were extracted with total RNA via the TRIzol method, and the RNA was reverse transcribed into cDNA according to the reverse transcription kit instructions. Glyceraldehyde-3-phosphate dehydrogenase (GAPDH) was selected as the housekeeping gene, and quantitative real-time PCR (qRT-PCR) primers were designed using Primer Express software (Applied Biosystems Incorporated, Foster City, CA, USA; Table 2). qRT-PCR was performed with ChamQTM SYBR® qPCR Master Mix (Vazyme, Nanjing, China) to detect the relative expression of the target genes using the primer sequences in Table 2. The PCR amplification was performed in a LightCycler96® (Roche Diagnostics GmbH, Mannheim, Germany) with cycling conditions as follows: 95°C for 30 s, 40 cycles of 5 s at 95°C, and 30 s at 55°C followed by a dissociation curve analysis step. All reactions were performed in triplicate.

**Table 2:** The primer sequences of qRT-PCR.

**Primer name**	**Primer sequence (5^**′**^-3^**′**^)**	**Product size (bp)**	**GenBank no**.
qHMGB2-F	TGAACACCGTCCAAAAATCA	125	XM_027456410.1
qHMGB2-R	ACCATACGAACAGAAGGCTG		
qTLR2- F	AAGAAAATGGAGCTGCTGGA	231	KX687002.1
qTLR2-R	GAAAAACACAGCGCAGATCA		
qTLR3-F	GAGTTTCACACAGGATGTTTAC	201	KU949327.1
qTLR3-R	GTGAGATTTGTTCCTTGCAG		
qTLR4-F	ACCCATTGTCACCAACATCATC	195	NM_001310413.1
qTLR4-R	TGCCTCAGCAAGGTCTTATTCA		
qRIG-I-F	GCTACCGCCGCTACATCGAG	224	KP981415.1
qRIG-I-R	TGCCAGTCCTGTGTAACCTG		
qIL-1β-F	TCATCTTCTACCGCCTGGAC	149	DQ393268.1
qIL-1β-R	GTAGGTGGCGATGTTGACCT		
qdIL-6-F	TTCGACGAGGAGAAATGCTT	150	JQ728554.1
qdIL-6-R	CCTTATCGTCGTTGCCAGAT		
qTNF-α-F	GAAGGGAATGAACCCTCCTC	89	EU375296.1
qTNF-α-R	CAGGTTGCTGCACATACACC		
qIFN-α-F	TCCTCCAACACCTCTTCGAC	232	KF731866.1
qIFN-α-R	GGGCTGTAGGTGTGGTTCTG		
qIFN-β-F	AGATGGCTCCCAGCTCTACA	210	KM035791.2
qIFN-β-R	AGTGGTTGAGCTGGTTGAGG		
qIFN-γ-F	GCTGATGGCAATCCTGTTTT	247	KF746067.1
qIFN-γ-R	GGATTTTCAAGCCAGTCAGC		
qOAS-F	TCTTCCTCAGCTGCTTCTCC	187	KY775584.1
qOAS-R	ACTTCGATGGACTCGCTGTT		
qPKR-F	AATTCCTTGCCTTTTCATTCAA	118	KR025553.1
qPKR-R	TTTGTTTTGTGCCATATCTTGG		
qMx-F	TGCTGTCCTTCATGACTTCG	153	KR025554.1
qMx-R	GCTTTGCTGAGCCGATTAAC		
qGAPDH-F	ATGTTCGTGATGGGTGTGAA	176	GU564233.1
qGAPDH-R	CTGTCTTCGTGTGTGGCTGT		
qDTMUV-F	CGCTGAGATGGAGGATTATGG	225	KP096415.1
qDTMUV-R	ACTGATTGTTTGGTGGCGTG		
qNDRV-F	TGAGTGGCTGGGAACTGT	233	JX826587.1
qNDRV-R	CCATAAAGGAAGCAGAAG		
qDPV-F	GCTTCACCTGCCCGGTCACAA	113	JQ647509.1
qDPV-R	CCACTGTCGGCACATCTAGCA		

### RNA Interference

Three interfering RNA-targeting HMGB2 sequences were purchased from GenePharma (Shanghai, China); the sequences of the synthesized small interfering RNA (siRNA) are shown in Table 4. Three interfering RNA and negative control (NC) interfering RNA were transfected into DEF cells using Lipofectamine 2000 (Invitrogen, Carlsbad, CA, USA). The DEF cells were seeded in 6-well plates and transfected with 2 μg/well of siRNA. Their interference efficiencies were analyzed by q-RT-PCR after 36 h of transfection.

**Table 3:** Reference sequences information.

**Species**	**GenBank accession number**
*Alligator sinensis*	XM_006022231.3
*Anas platyrhynchos*	XM_027456410.1
*Anser cygnoides*	XM_013190319.1
*Callorhinchus milii*	NM_001292834.1
*Chelonia mydas*	XM_007067058.2
*Columba livia*	XM_005510908.2
*Equus caballus*	XM_023636290.1
*Gallus*	NM_205486.1
*Geospiza fortis*	XM_005415550.2
*Gorilla*	XM_004040632.3
*Homo*	NM_001130688.1
*Lctalurus punctatus*	XM_017463391.1
*Mus muculus*	NM_001363443.1
*Oryctolagus cuniculus*	XM_002709471.3
*Papio Anubis*	XM_003899366.5
*Pelodiscus sinensis*	XM_003899366.5
*Python bivittatus*	XM_007430032.3

**Table 4:** Sequences of small interfering RNA.

**SiRNA**	**Sense sequence (5^**′**^-3^**′**^)**	**Antisense sequence (5^**′**^-3^**′**^)**
Si-duHMGB2-1	GGGAGACAAAGCUC GUUAUTT	AUAACGAGCUUUGUCUCCCTT
Si-duHMGB2-2	GGCCUGCAGGAUCU AAGAATT	UUCUUAGAUCCUGCAGGCCTT
Si-duHMGB2-3	GCAUCCUGGCUUG UCUAUUTT	AAUAGACAAGCCAGGAUGCTT
Si-NC	UUCUCCGAACGUGU CACGUTT	ACGUGACACGUUCGGAGAATT

### Dual-Luciferase Reporter Assay

DEF cells were cultured overnight at 37°C in 24-well plates. When the cells reached 80% confluence, the expression plasmid or empty vector (500 ng/well), reporter plasmid (pGL3-IRF7-Luc and pGL3-NF-κB, 100 ng/well) (4), and pRL-TK plasmid (50 ng/well; Promega, Madison, WI, USA) were co-transfected into DEF cells by Lipofectamine 2000 (Invitrogen, Carlsbad, CA, USA). We constructed the reporter plasmids (pGL3-IRF7, pGL3-NF-κB-luc) as follows using the promoter sequence of the avian (chicken) for IFN-β: CCT CCA GTA CAG CCA CCA CAT GGT CTC ACC TTG CCA GAC TCA AGA GAA GCC TGA AGG AAA AAA GCA AAT AGA AAG CAA AAC GAA AAA TGG AAA CAA GGG AAT TCT CTC TAC ATA ATG ATG AAA AGA AAC ATG CAA CAT CTC ATA AAG CTG GCC TCA CTG CAA CAC CCC AAAC. The chicken IRF-7 (chIRF-7) binding positive regulatory domains were predicted by the TFSEARCH: Searching Transcription Factor Binding Sites. The pGL3-chIRF-7-Luc contains four copies of the IRF-7-positive regulatory domain motif of the chicken IFN-β promoter upstream of a luciferase reporter gene (sequence: TTC ACT TTC AAT A). According to the manufacturer's instructions (Promega, Madison, WI, USA), luciferase activity was measured at 48 h post-transfection (hpt) using the dual-luciferase reporter assay system.

### Statistical Analysis

The relative expression of genes was calculated using the 2^−ΔΔCT^ method during qRT-PCR data processing. The data in the experiment were evaluated with Excel software to determine the means and SE of each group of data. SPSS19.0 software was used to analyze the difference between each group of data. The data with only two groups were tested via an independent sample student's *t-*test with *P* < 0.05 indicating a significant difference.

## Results

### Characterization of duHMGB2

**Figure 1:**
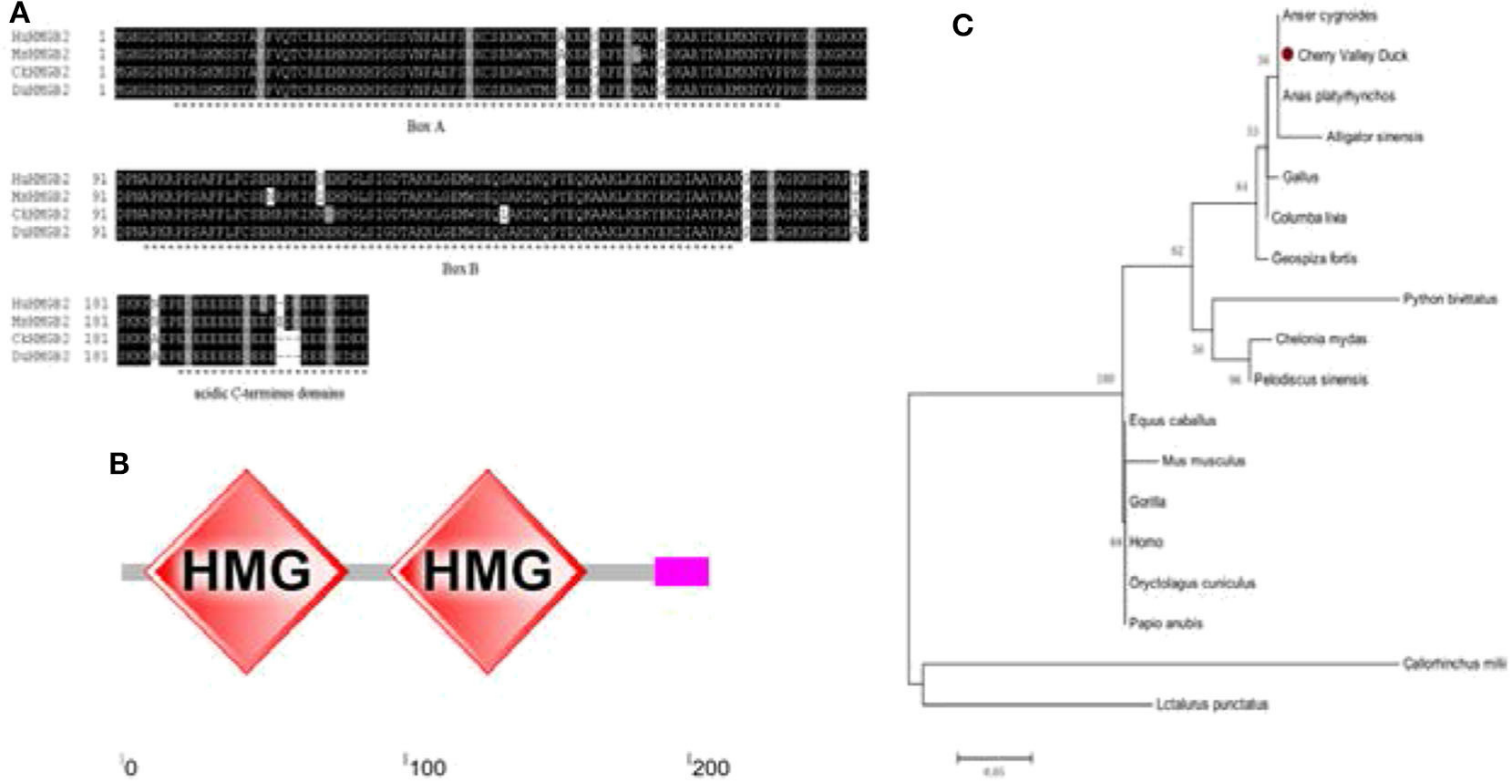
Characterization of duHMGB2. **(A)** Amino acid multiple sequence alignment of duHMGB2. Amino acid alignment was determined by the Clustal X program and edited with Boxshade. HMGB2 sequences are shown for the human (Hu), mouse (Mu), chicken (Ch), and Cherry Valley duck (Du). Black shading indicates amino acid identity and gray shading indicates similarity (50% threshold). **(B)** The protein motifs of duHMGB2 were analyzed using SMART. **(C)** The phylogenetic tree is based on duHMGB2 homologs. The neighbor-joining tree was generated using MEGA 5.0, and a 1,000-replicate bootstrap analysis was performed. The scale bar is 0.05. The GenBank accession numbers for each species are shown in Table 3.

The cloning primers were designed with reference to the duck's HMGB2 predicted sequence (GenBank accession number: XM_027456410.1) in NCBI, and the *HMGB2* CDs sequence was amplified from the spleen of healthy Cherry Valley ducks using the primers (Table 1). Figure 1A shows that the coding region of *duHMGB2* contains an ORF composed of 624 nucleotides encoding 207 amino acids (aa). The accession number of this sequence on GenBank is MT598189. The amino acid sequence of duHMGB2 was predicted and analyzed by SMART software, and it presented the typical structure of HMGBs: two functional domains that bind to DNA and an acidic C-terminus domain (183–202aa) (Figure 1B). Box A (3–75aa) is located at the N-terminus, and box B (89–159aa) is located in the middle of the molecule. The phylogenetic tree of *HMGB2* was constructed with MEGA5.0 software. The duHMGB2 is marked with a red circle in the phylogenetic tree in Figure 1C. The duHMGB2 phylogenetic tree is constructed from the aa sequences (CDs) of 18 different species including mammals, birds, and reptiles. The phylogenetic tree shows that *duHMGB2* has the highest homology with the Mallard and Prowl duck species predicted on NCBI and has lower homology with mammals and reptiles. Analysis with DNAMAN software found that *duHMGB2* had 100% sequence homology with *Anas platyrhynchos*, 94.39% homology with the *Gallus*, 82.86% homology with *Homo sapiens*, and 82.46% homology with *Mus musculus*. This is consistent with the phylogenetic tree.

### Distribution of duHMGB2 in Healthy Duck Tissues

**Figure 2:**
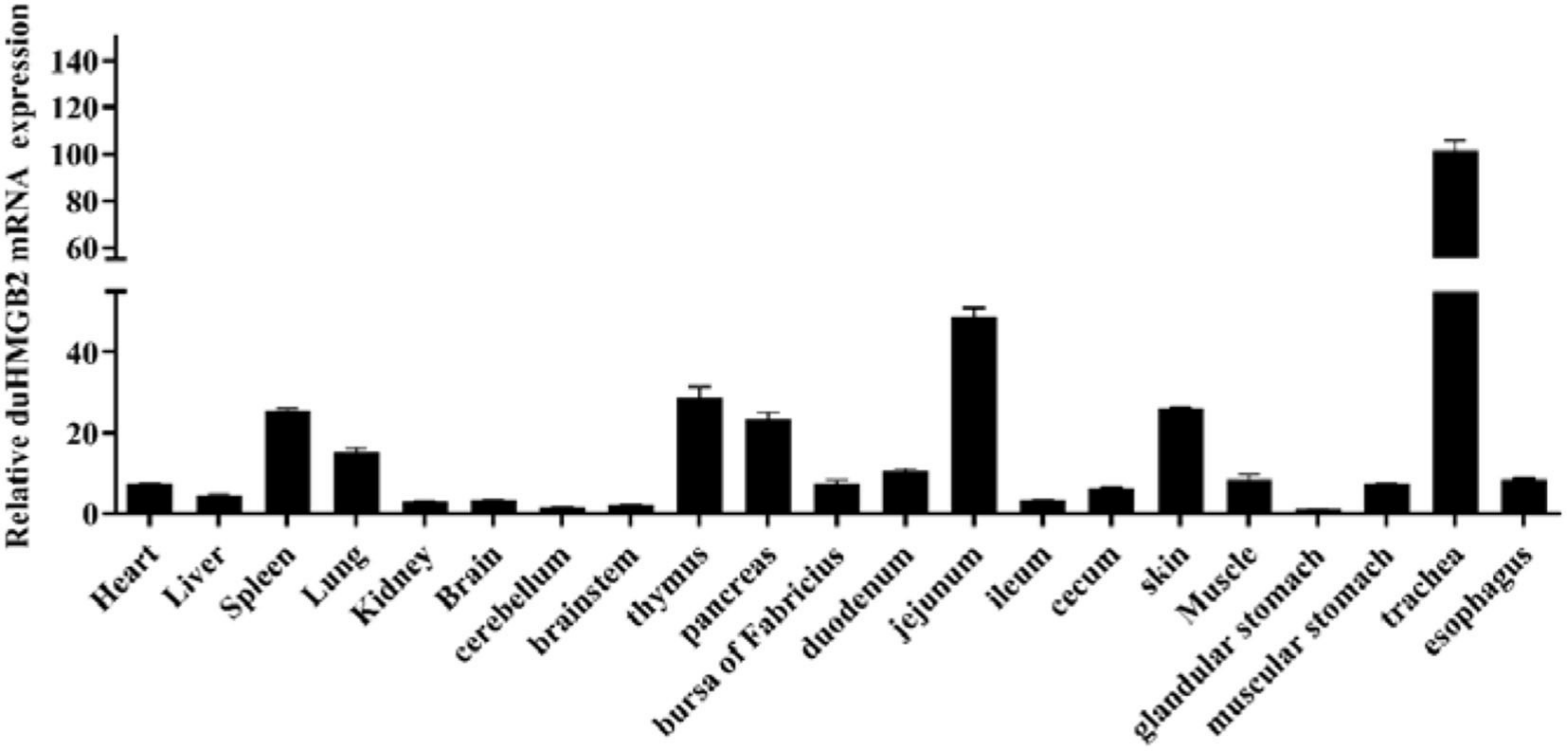
Tissue distribution of duHMGB2 transcripts in healthy Cherry Valley ducks. The relative mRNA levels were normalized to the expression of the GAPDH gene from various tissues. Each result represents the expression level of duHMGB2 relative to the glandular stomach in the test tissue. Values are the mean value ± SE of three experiments.

The distribution of the *duHMGB2* gene in 21 healthy duck tissues was analyzed by qRT-PCR. Figure 2 shows that while duHMGB2 was expressed in all 21 tested tissues, there are obvious differences in the expression levels of different tissues. The expression level of duHMGB2 in the trachea was 101.5-fold higher than the expression of duHMGB2 in the glandular stomach. The expression of duHMGB2 was weak in the cerebellum, brainstem, and kidney. These results indicated that duHMGB2 was extensively distributed in the host and may play an indispensable role in the host's innate immune response.

### Expression of duHMGB2 in Virus-Infected Ducks

**Figure 3:**
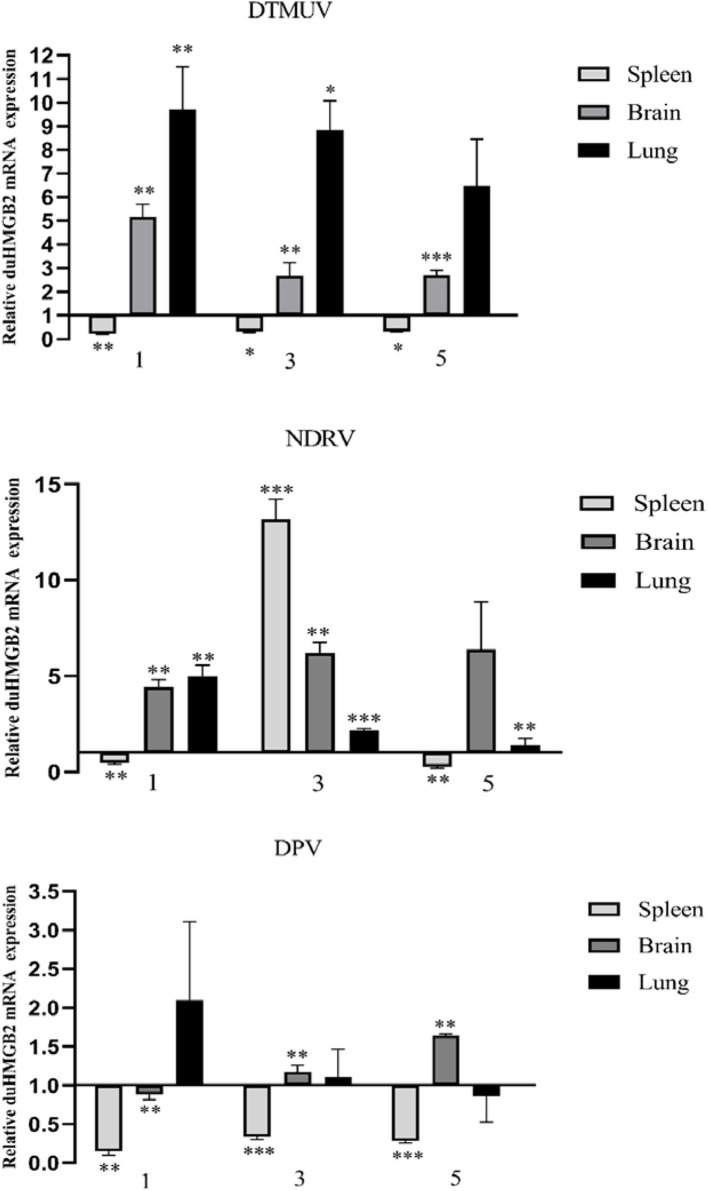
Expression of duHMGB2 in the three tissues and serum of virus-infected ducks. The relative mRNA levels were normalized to the expression of the GAPDH gene from various tissues. Each result represents the expression level of duHMGB2 relative to the glandular stomach in the test tissue. Values are shown as the mean value ± SE of three experiments. *P < 0.05; **P < 0.01; ***P < 0.001.

To explore whether duHMGB2 is involved in the host antiviral immune response to virus-infected ducks, the expression of duHMGB2 mRNA in the spleen, brain, and lung was detected by qRT-PCR at 1, 3, and 5 days post-infection (dpi) (Figure 3). Following DTMUV infection in ducks, duHMGB2 was significantly up-regulated at 1 dpi in the lungs (9.7-fold, *P* < 0.01). The upward trend was then reduced; the sample was finally up-regulated by 6.5-fold at 5 dpi. During NDRV infection, duHMGB2 was significantly up-regulated in the spleen at 3 dpi (13.2-fold, *P* < 0.001). The expression level of *duHMGB2* in the lung was highest at 1 dpi (5-fold, *P* < 0.001), and the expression level in the brain was up-regulated with an increase of 6.4-fold at 5 dpi. However, the up-regulation of *duHMGB2* expression in the test organs was compared to control ducks was not very significant during DPV infection.

### Subcellular Localization of duHMGB2 in DEF Cells

**Figure 4:**
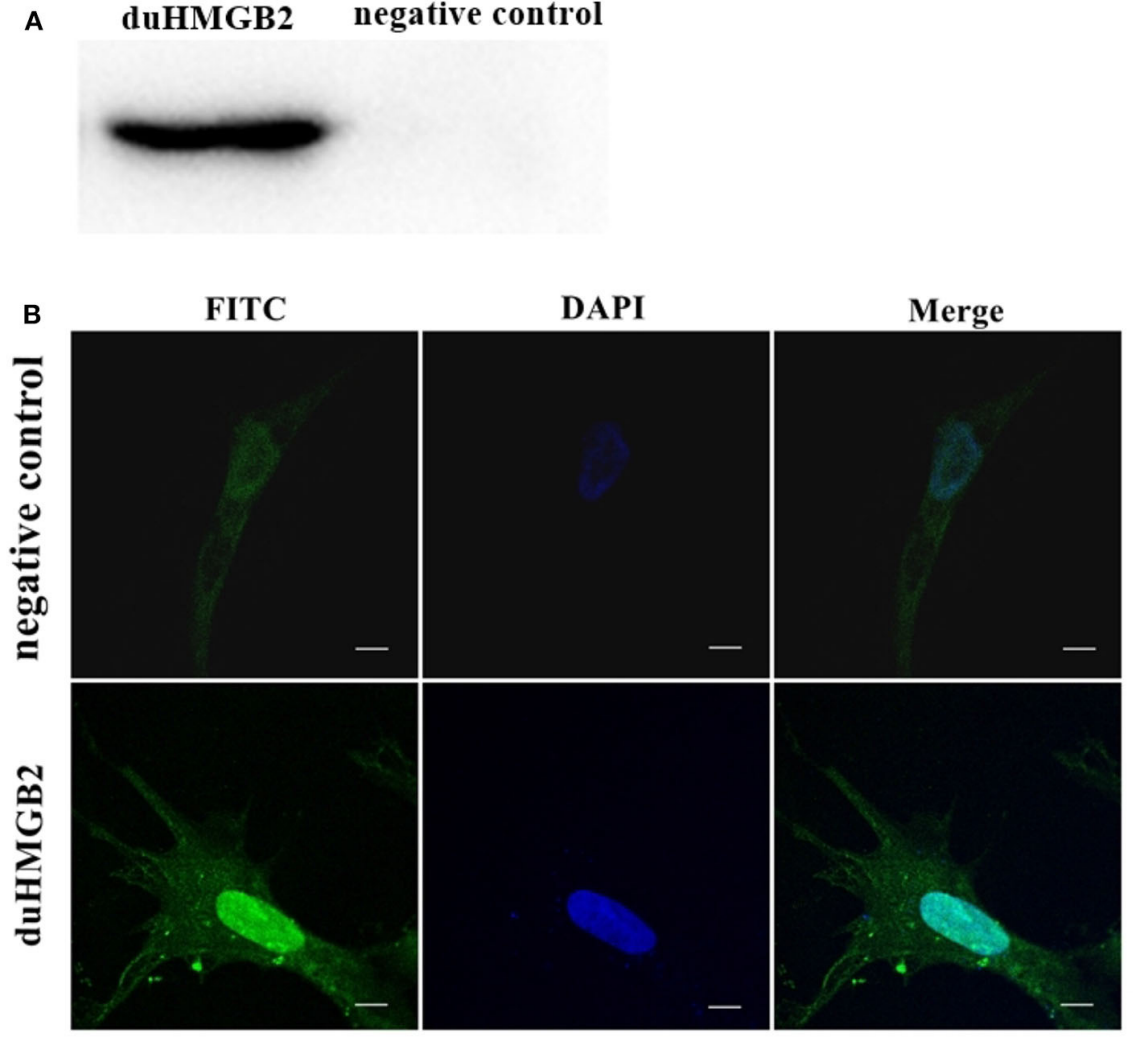
The recombinant duHMGB2 plasmid is mainly expressed in the nucleus of DEF cells. **(A)** Western blot detection after the recombinant plasmid pcDNA3.0 (+)-duHMGB2-Flag was transfected into DEF cells. The left lane is the control group, and the right lane is the experimental group. **(B)** Indirect immunofluorescence of duHMGB2 subcellular localization. The primary antibody is mouse anti-Flag antibody, and the secondary antibody is FITC-labeled goat anti-mouse IgG antibody (green). Nuclei were counterstained with DAPI (blue). All scale bars represent 10 μm.

Figure 4A shows the western blotting results, which indicate that the duHMGB2 recombinant eukaryotic expression plasmid was successfully expressed in DEF cells. Furthermore, the confocal microscopy data showed that duHMGB2 was mainly located in the nucleus of DEF cells, and only a small part was located in the cytoplasm (Figure 4B).

### Spatial and Temporal Distribution of duHMGB2 Localized by IFA and Confocal Microscopy in DEF Cells

**Figure 5:**
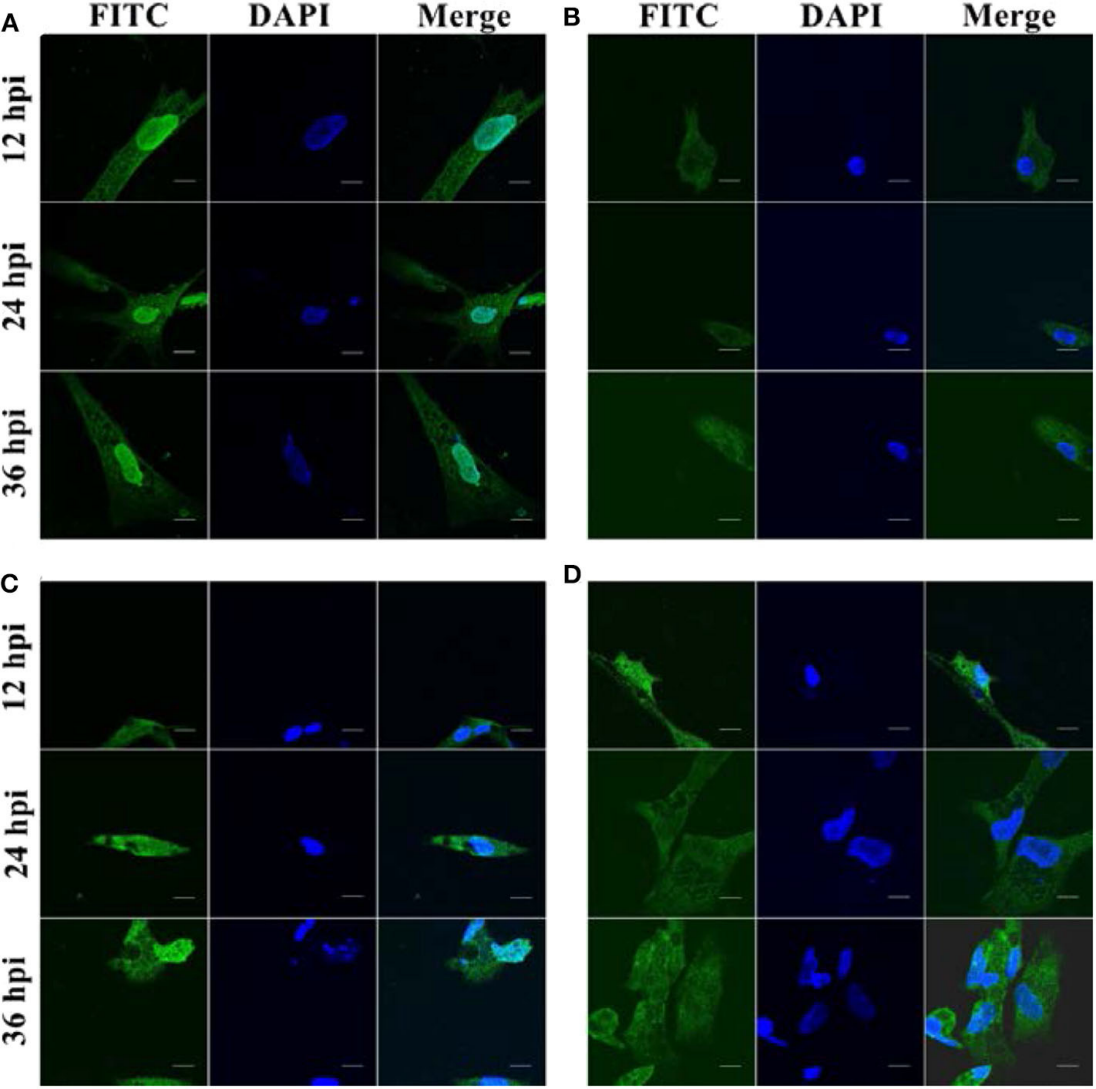
Spatial and temporal distribution of duHMGB2 in DEF cells. **(A)** Control group: Immunofluorescent imaging of DEF cells transfected with pcDNA3.0(+)-duHMGB2-Flag plasmid. **(B–D)** Viruses-infected groups: **(B)** DEF cells transfected with pcDNA3.0(+)-duHMGB2-Flag plasmid and infected with 10 TCID_50_/mL DPV; **(C)** DEF cells transfected with pcDNA3.0(+)-duHMGB2-Flag plasmid and infected with 10 TCID_50_/mL DTMUV; **(D)** DEF cells transfected with pcDNA3.0(+)-duHMGB2-Flag plasmid and infected with 10 TCID_50_/mL NDRV. duHMGB2 appears in green, and DAPI in blue. All scale bars represent 10 μm.

We next investigated the sub-localization of duHMGB2 in DEF cells after infection with DPV, DTMUV, and NDRV. DEF cells were treated with the three viruses or PBS after transfection with pcDNA3.0(+)-duHMGB2-Flag plasmid. We found that duHMGB2 transferred to the cytoplasm after the viral infection as seen by IFA and confocal microscopy (Figures 5B–D). Vacuoles appeared in the cells over time, and the number of vacuoles was the largest in cells at 36 h post-infection (hpi) (Figure 5).

### The Innate Immune Response in DEF Cells With Overexpression of duHMGB2

**Figure 6:**
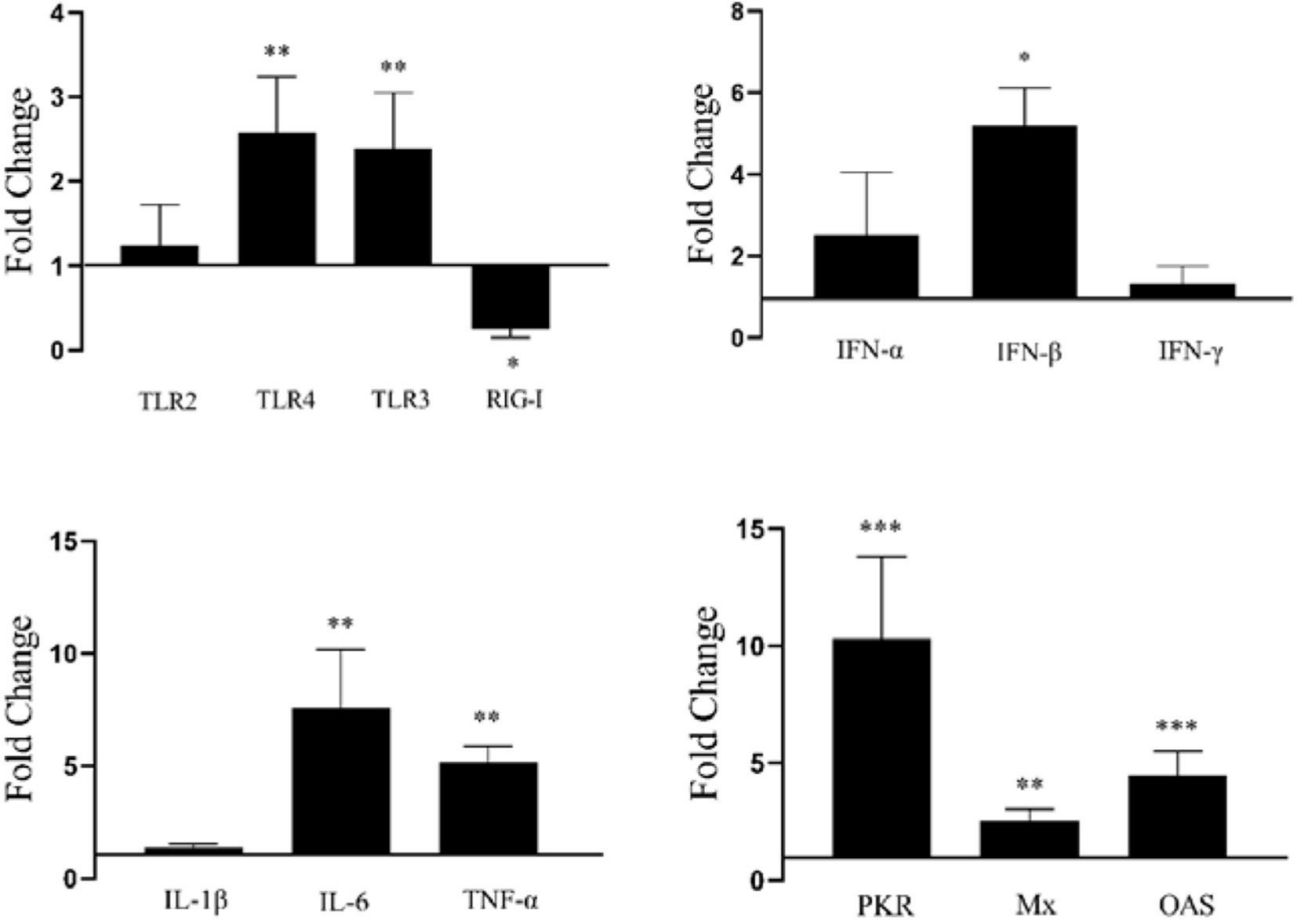
Overexpression of duHMGB2-induced changes in immune genes. The 2^−ΔΔCT^ method with GAPDH as the internal reference gene was used to calculate the change in gene expression. The data were expressed as the mean ± SE of three experiments. Student's *t*-test used to analyze the differences between two groups. *P < 0.05; **P < 0.01; ***P < 0.001.

To further study duHMGB2-induced innate immune responses, the pcDNA3.0(+)-duHMGB2-Flag or pcDNA3.0(+)-Flag (empty vector) were transfected into DEF cells. At 48 h after transfection (hpt), the cells were collected and examined for gene expression. The mRNA expression of *TLR3, TLR4, OAS, PKR, Mx, IFN-*β, *TNF-*α, and *IL-6* were significantly upregulated with the overexpression of duHMGB2 in DEF (Figure 6). The ISGs including *Mx, PKR*, and *OAS* were significantly upregulated, and the most obvious increase was in *PKR* (10.3-fold, *P* < 0.001). The expression levels of proinflammatory cytokines (*TNF-*α, *IL-1*β and *IL-6*) were upregulated, and the mRNA expression levels of TNF-α and IL-6 were significantly increased by 5.2-fold (*P* < 0.01) and 7.2-fold (*P* < 0.01), respectively. These changes indicated that duHMGB2 overexpression could induce the production of antiviral proteins and proinflammatory cytokines.

### Overexpression of duHMGB2 Activates the NF-κB Signaling Pathway

**Figure 7:**
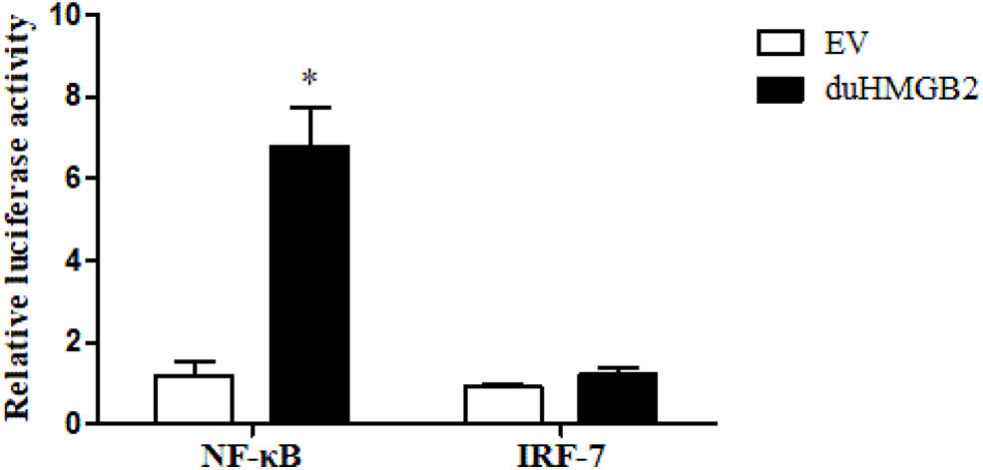
duHMGB2 activates NF-κB promoter activity. The promoter activity was detected using the dual luciferase reporter gene assay. The control group was set to 1 to calculate the fold change relative to the firefly luciferase activity. Student's *t-*test was used to analyze the differences between the two groups. *P < 0.05.

PcDNA3.0(+)-duHMGB2-Flag and empty vector (control group) plasmids were co-transfected with reporter plasmids (pGL3-IRF7, pGL3-NF-κB-luc) with pRL-TK (normalization) to detect the effect of duHMGB2 on downstream gene expression. Cells were harvested after 48 hpt, and luciferase activity was measured. Figure 7 shows that overexpression of duHMGB2 in DEF cells had no significant effect on the IRF-7 promoter activity. However, duHMGB2 significantly activated NF-κB luciferase activities vs. empty vectors (5.7-fold at 48 hpt, *P* < 0.05).

### The Antiviral Ability of duHMGB2 in Infected DEF Cells

PcDNA3.0-duHMGB2-Flag (experimental group) and pcDNA3.0-Flag (control group) were transfected into DEF cells. After 24 h, these cells were infected with 10 TCID_50_/mL virus (NDRV, DPV, and DTMUV), and the culture supernatants were collected at 12, 24, 36, and 48 hpi. The relative fluorescent PCR method was used to compare the changes of the expression levels of related viral genes in the experimental group relative to the control group.

**Figure 8:**
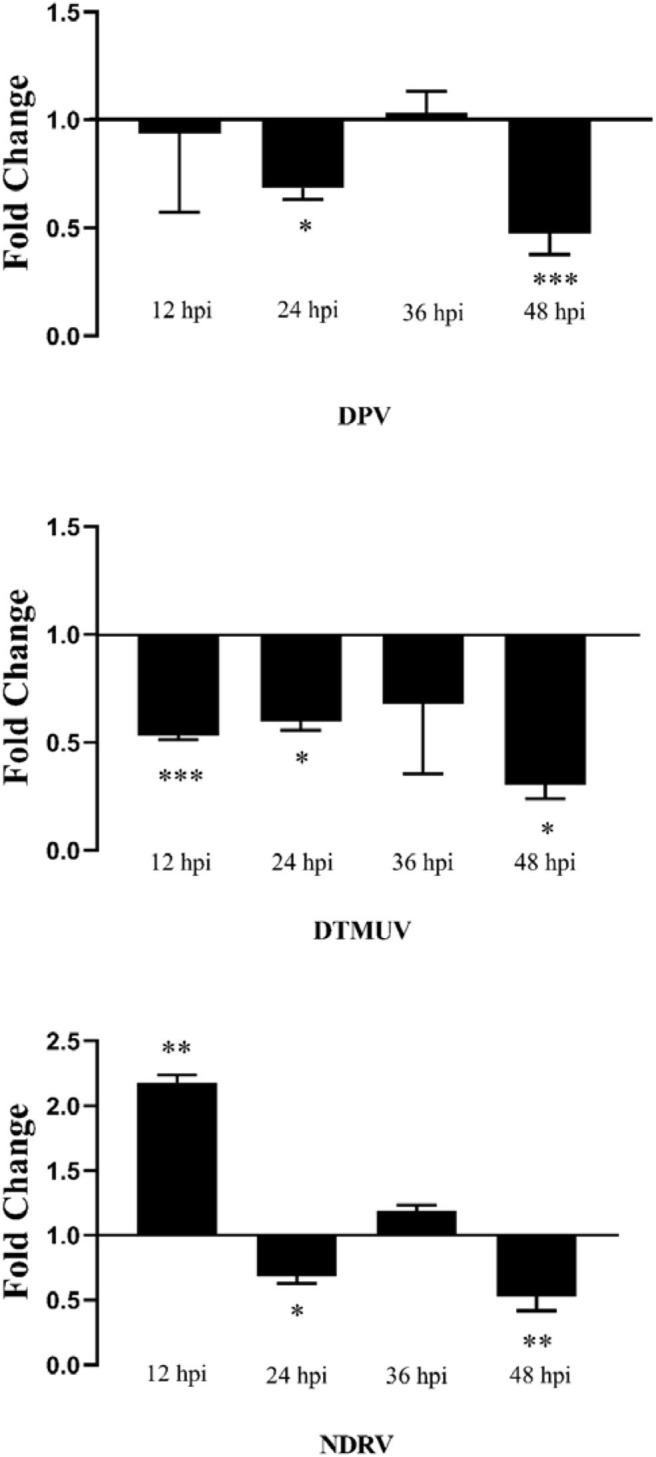
Antiviral assay of duHMGB2 in DEF cells. Fold-changes in DPV, DTMUV, and NDRV gene expression were calculated using the 2^−ΔΔCT^ method with GAPDH serving as a normalization gene. Data represent the means ± SE of three experiments. *P < 0.05; **P < 0.01; ***P < 0.001.

Figure 8 shows that the expression level of viruses (NDRV, DTMUV, and DPV) in DEF cells transfected with pcDNA3.0-duHMGB2-Flag was lower than that of the control group during the experimental period. These results indicate that duHMGB2 has a good spectrum of antiviral effects. The antiviral effect of duHMGB2 on DTMUV was more pronounced. For example, the expression of DTMUV in the experimental group was reduced by 3.4-fold vs. the control group at 48 hpi (*P* < 0.05).

### Analysis of Antiviral Ability After Knocking Down duHMGB2

**Figure 9:**
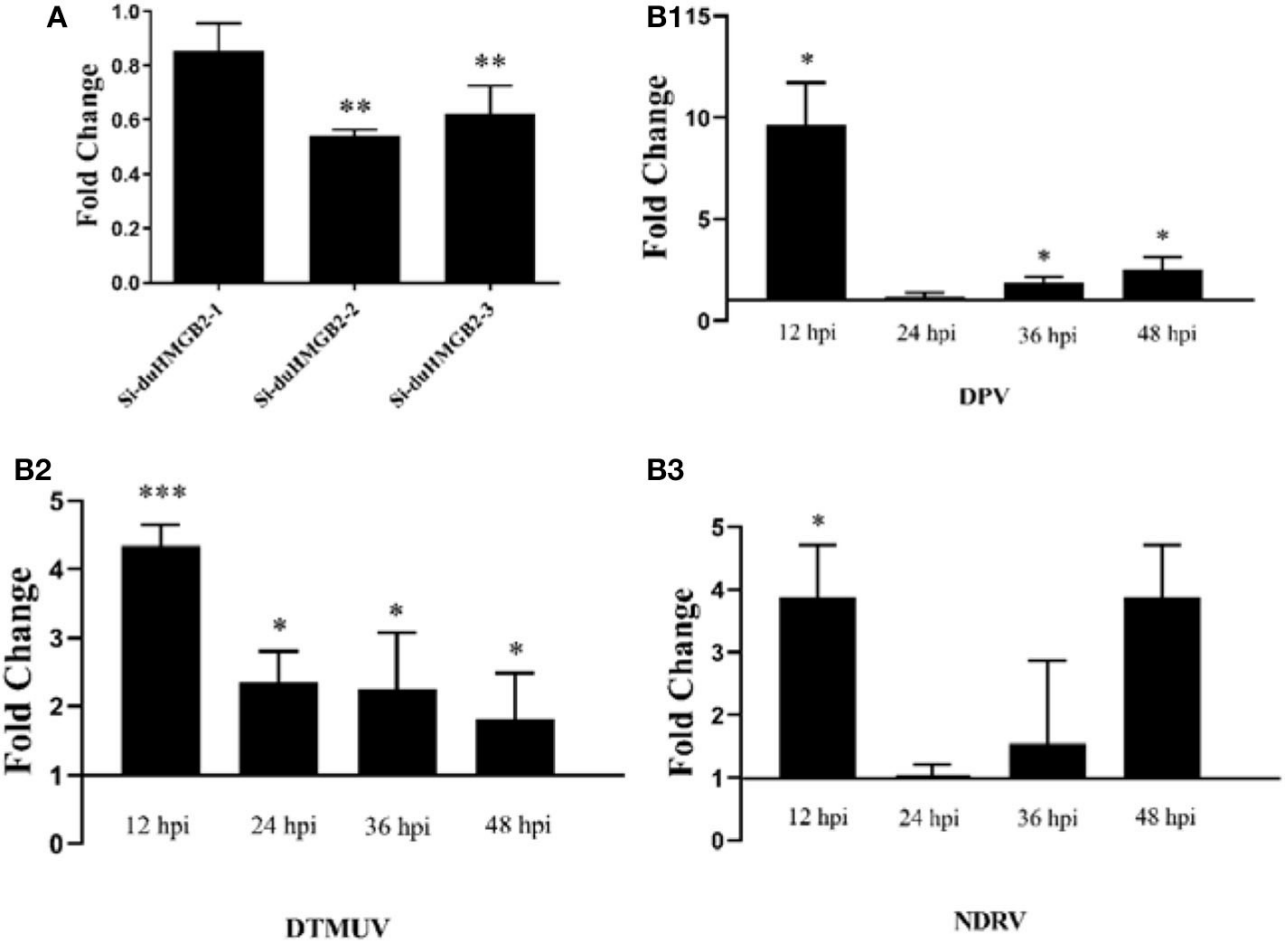
**(A)** The RNA interference efficiency of duHMGB2-2 is 50.4%. siRNA for duHMGB2 and siRNA control were transfected, and DEF cells were cultured for 36 h. The duHMGB2 expression levels were normalized to the GAPDH gene and calculated using the 2^−ΔΔCt^ method. Data are represented as the mean value ± SE of three experiments. **(B)** The duHMGB2 knockdown increases the replication of DPV, DTUMV, and NDRV. The virus released into the supernatant was measured by qRT-PCR 36 h after transfection with siRNA. Data was calculated with the 2^−ΔΔCt^ method using GAPDH RNA as the reference gene. Two-tailed Student's *t-*tests were used to analyze the differences between the two groups. Data are expressed as means ± SE of three independent experiments. *P < 0.05; **P < 0.01; ***P < 0.001.

Figure 9A shows that both Si-duHMGB2-2 and Si-duHMGB2-3 can significantly inhibit the expression of duHMGB2, and the interference efficiency of Si-duHMGB2-2 is higher. Therefore, Si-duHMGB2-2 was chosen for subsequent experiments. Si-NC and Si-duHMGB2-2 were transfected into DEF cells. After 36 h, cells were infected with 1 TCID_50_/mL virus (DPV, DTMUV, and NDRV). The results showed that the expression of DPV, DTMUV, and NDRV in the DEF cells experimental group increased at all time points vs. the control group (Figures 9B1–3). For example, at 12 hpi, the expression levels of DPV, DTMUV, and NDRV increased by 9.8-fold (*P* < 0.05), 4.3-fold (*P* < 0.001), and 3.8-fold (*P* < 0.05), respectively. These observations show that knocking down the expression of duHMGB2 in DEF cells increases the replication of DPV, DTMUV, and NDRV in DEF cells.

## Discussion

The HMGB family belongs to chromatin-associated non-histone proteins (17). HMGB1 and HMGB2 have been implicated in numerous cellular processes including proliferation, differentiation, DNA replication, recombination, repair, transcription, inflammation, tumor migration, and cell signaling (1, 8, 18, 19). The results show that duHMGB2 can activate the antiviral innate immunity of the host, and our results provide an explanation for the biological function of HMGB2.

Previous studies have shown that HMGB2 was present in all cultured cells and was abundant in the thymus. However, *HMGB2* expression changed as mice aged. In adult mice, HMGB2 is mainly expressed in the lymphoid organs and testes (20). Our results show that duHMGB2 was widely distributed in healthy duck tissues, and the duHMGB2 expression was highest in the trachea. This suggests that the expression of HMGB2 varies enormously across different tissues and species. These differences suggest that duHMGB2 protein may directly or indirectly participate in the host's natural antiviral immune response.

In host immunity, HMGBs respond to bacterial/viral PAMP challenge as a sensor (8). Once released into the extracellular environment, HMGBs behave as a sensor activating different PRRs such as TLRs and RLRs (7, 21, 22). TLRs and RLRs interact differently with TRIF, MyD88, and IPS-1 to phosphorylate IRF3/7 or NF-κB resulting in the expression of IFN-I and cytokines (23).

An in-depth study of HMGB2 has shown that HMGB2 plays a role in innate immunity and adaptive immune response, but there are relatively few studies on the innate immune role in host antiviral infection. Therefore, this study cloned the *duHMGB2* gene isolated from the spleen of a Cherry Valley duck and compared it with the *HMGB2* sequences across various species to detect the genetic relationships via a phylogenetic tree. The results showed that Cherry Valley duck *HMGB2* had the highest homology with mallard ducks followed by chickens; there was less homology with mammals and reptiles.

The duHMGB2 was mostly expressed in the nucleus and transferred to cytoplasm after viral infection. Bonaldi et al. (24) found that HMGB1 has two nuclear localization sequences and cellular localization of HMGB1 in monocytes can be regulated by a nuclear re-shuttling mechanism and (de)acetylating activities that switch a chromatin protein into a cytokine in response to inflammatory stimuli.

Previous studies have shown that extracellular HMGB1 binds to TLR4 and leads to the activation of NF-κB mediated by MyD88 (25). Chicken HMGB1 is involved in NDV-induced NF-κB activation and the associated inflammatory response (26). qRT-PCR results show that overexpression of duHMGB2 in DEF cells induced a strong expression of *TLRs* (*TLR3* and *TLR4*) and anti-viral molecules (PKR, OAS, and Mx) as well as INF-I and pro-inflammatory cytokines (*TNF-*α, *IL-1*β, and *IL-6*). This release indicates that duHMGB2 can play an important role in the innate immunity of the duck host response against virus infection and can activate the host immunity and enhance the antiviral effects. We also show that duHMGB2 overexpression in DEF cells can significantly activate NF-κB luciferase activities. However, the functional cooperation between these species requires further research.

Previous research has shown that *Cynoglossus semilaevis* HMGB2 possesses immunoregulatory properties that promote resistance against bacterial and viral infection (27). Two sets of experiments were designed to further analyze whether duHMGB2 has antiviral replication effects on duck disease virus: a duHMGB2 overexpression experimental group and a duHMGB2 knockdown experimental group. We then analyzed the level of duck disease virus gene expression in the experimental group and the control group. We used positive and negative directions to verify that duHMGB2 had an antiviral replication effect on DPV, DTMUV, and NDRV. This may be because duHMGB2 activated the innate immune system; the specific mechanism requires further research and discussion.

In summary, *duHMGB2* is the homolog of *HMGB2* and was cloned and characterized from the Cherry Valley duck. We found that it has extensive *in vivo* expression and is involved in the immune response to DPV, DTMUV, and NDRV infection. *In vitro* reconstitution experiments showed that duHMGB2 can activate the host innate immune signaling pathways that govern IFN-mediated antiviral immune response. These results offer important clues for understanding the role of duHMGB2 in innate immunity; duHMGB2 is expected to provide potential therapeutic targets for eradication of infectious diseases in ducks.

## Data Availability Statement

The datasets presented in this study can be found in online repositories. The names of the repository/repositories and accession number(s) can be found in the article/supplementary material.

## Ethics Statement

The animal study was reviewed and approved by All animal experiments were performed in accordance with the recommendations of the Shandong Agricultural University Animal Care and Use Committee. The approval number for this study is SDAUA-2019-016.

## Author Contributions

TZ and XZ wrote the manuscript and performed the most of the experiments. ZS and GL collected the samples and extracted the sample RNA. XH and LW designed the study and polished the article. All authors contributed to the article and approved the submitted version.

## Conflict of Interest

The authors declare that the research was conducted in the absence of any commercial or financial relationships that could be construed as a potential conflict of interest.
